# Correlation between the Ability to Manipulate a Touchscreen Device and Hand Strength and Manual Dexterity among Community-Living Older Individuals

**DOI:** 10.3390/ijerph18179408

**Published:** 2021-09-06

**Authors:** Michal Elboim-Gabyzon, Alexandra Danial-Saad

**Affiliations:** 1 Department of Physical Therapy, Faculty of Social Welfare & Health Sciences, University of Haifa, Haifa 3498838, Israel; 2 Department of Occupational Therapy, Faculty of Social Welfare & Health Sciences, University of Haifa, Haifa 3498838, Israel; saadalexandra@gmail.com; 3 The Arab Academic College for Education in Israel, University of Haifa, Haifa 3498838, Israel

**Keywords:** hand function, older adults, assessment, touchscreen manipulation, touchscreen assessment tool, TATOO

## Abstract

Information regarding the relationship between the degree of hand function among the elderly as measured by traditional assessments and the ability to manipulate touchscreens is lacking. This study aimed to examine the correlation between the ability to manipulate a touchscreen device, as assessed using the touchscreen assessment tool (TATOO) (University of Haifa, Israel & Universetiy of Bologna, Italy), and hand strength and manual dexterity among independent community-living older individuals. Thirty-four community-living older adults (average age 79.4 ± 6.7 years) participated in single-session assessments lasting 45 min each. The assessment included hand strength measurement using the manual hand dynamometry and hydraulic pinch gauge, a functional dexterity test (FDT), and TATOO. No significant correlations were observed between most of the TATOO items (22 out of 26) and handgrip strength, pinch strength, and FDT results. Moderately significant correlations were demonstrated between the number of drag attempts in the “Drag to different directions” task and handgrip strength and manual dexterity (*r* value: −0.39, *p* value: 0.02; *r* value: 0.36, *p* value: 0.04, respectively). In addition, a moderately significant correlation was noted between the number of double taps and manual dexterity (*r* value: 0.32, *p* value: 0.07). These results indicate that more complex gestures that require greater accuracy (dragging task) or rapid movements (double tapping) are related to hand strength and manual dexterity. These results suggest that the manual gestures necessary for touchscreen operation entail unique and specific capabilities that are generally not captured by traditional tools. The clinical implication is that the hand function assessment toolbox should be expanded. Tools such as the TATOO should be used to capture skills required for touchscreen manipulation in the context of the modern digital milieu.

## 1. Introduction

Manual hand function decreases with normal aging due to the progressive degenerative processes of the musculoskeletal, vascular, and nervous systems [1,2,3]. The impact of these changes has a deleterious effect on the performance of basic and instrumental activities of daily life (BADL, IADL) [1,2]. The musculoskeletal component of hand function is usually assessed by measuring hand grip strength using manual dynamometry and pinch strength using the hydraulic pinch gauge [4,5,6]. Furthermore, hand grip and knee extension strength are frequently used to objectively quantify age-related decline in body muscle strength (dynapenia) [4,7,8]. In addition, hand grip strength is considered an important health indicator, as it correlates with geriatric adverse conditions, such as frailty, coronary heart disease, falls, disability, and mortality [9]. Hand function assessments often include the evaluation of age-related impairments in the sensory system and the interaction between the sensory and motor systems [2,10,11]. These impairments are reflected in decreased tactile sensibilities, reduced fine motor coordination, reduced manual speed, and increased response time [2,10,11]. Intactness of the sensory-motor system is manifested in manual dexterity, defined as the ability to manipulate objects with the hands [12]. Assessment of manual dexterity evaluates the quality, accuracy, ease, and speed of accomplishing manual tasks [13]. Several common tests, including the Jebsen Taylor test of hand function (JTHFT) [14] and the functional dexterity test [12], are used by clinicians to measure manual and finger dexterity. Previous studies have demonstrated a significant positive correlation between hand grip strength and dexterity and the performance of daily life activities among the elderly [15,16,17].

In addition, hand function among the elderly can be evaluated by assessing the actual performance of daily life activities that require hand movements [1,18]. Such assessments may involve observation of performance or self-reports utilizing validated questionnaires [1,18]. Furthermore, modern technologies can be utilized to quantify various aspects of hand movements. For instance, Jarque-Bou et al. [19] conducted a kinematic analysis among the general population of 26 representative daily activities that require the use of hands, such as opening and closing zippers, applying toothpaste to a toothbrush, taking a pen from a table, writing their name, and putting down the pen.

Independence and participation in the present digital world rely heavily on the ability to manipulate touchscreen devices, as many daily activities, such as communication via cellular phones, banking, and health services, require the use of touchscreen devices. However, the capabilities of elderly individuals to utilize this technology have received limited attention. For instance, hand activities analyzed by Jarque-Bou et al. [19] included only one item relevant to the digital environment—writing using a keypad—while other aspects relevant to the use of touchscreen interfaces were not addressed among the elderly.

The touchscreen assessment tool (TATOO) is a software application developed by Daniel-Saad and Chiari [20] to evaluate the ability to manipulate a touchscreen. It consists of six tasks providing information on the performance of different functional components required to use a touchscreen effectively, such as tapping, pinching, and dragging [20]. Elboim-Gabyzon et al. [21] demonstrated that the TATOO can be used to assess fine motor skills necessary in today’s technological environment, as it poses good discriminative validity and usability among older adults. A previous study used the TATOO tool to compare touchscreen performance among 28 older adults (aged 81.9 ± 4.2 years) living in independent communities and 25 healthy middle-aged adults (aged 53.4 ± 5.9 years) [21]. The results indicated that older adults had poorer temporal performance and were less accurate in most tasks when compared with the middle-aged group, such as requiring more time to complete tasks and more taps to achieve the desired goal. This pattern was more noticeable for more complex goal-oriented manual gestures, such as double-tapping, and gestures that involved nonlinear movement of the finger while maintaining constant pressure on the screen [21]. Furthermore, the study demonstrated that previous experience in manipulating a smartphone did not affect performance among older adults [21]. 

However, it is unclear to what degree the ability to manipulate a touchscreen is correlated with traditional hand function assessments tools. Therefore, this study aimed to assess and compare hand function among the elderly using traditional assessment tools and the TATOO. The objective of this study was to examine the correlation between the ability to manipulate a touchscreen device, as assessed using the TATOO, and hand grip strength, pinch strength, and FDT among independent community-living older individuals.

## 2. Materials and Methods

This observational study enrolled 34 community-living older adults (23 women and 11 men). The participants were aged 65 or older with an average (standard deviation) age of 79.4 (6.7) years. The participants were recruited from two senior citizen centers. This study was part of a larger ongoing study designed to validate the TATOO as an assessment tool for assessing specific motor skills needed to operate touchscreens among elderly adults from various culture backgrounds and health statuses. The study was approved by the ethical review board of the University of Haifa (protocol code 18/366). All participants provided written informed consent before participating in the assessment after a detailed explanation of the study objectives and design was provided. 

The inclusion and exclusion criteria were detailed in the previous publication [21]. Eligibility was verified in face-to-face interviews based on a structured questionnaire. The study included elderly individuals living independently in the community, able to walk independently without assistive devices, and able to follow simple commands. 

### 2.1. Instruments

Data on age, gender, known health and medical conditions, functional level, use of a walking device, and experience with touchscreen devices were collected. The assessments for hand grip strength, pinch strength, and manual dexterity, and the touchscreen assessment tool (TATOO) were performed in a random order.

#### 2.1.1. Hand Grip and Pinch Strength

We assessed three types of grip strengths that are most commonly applied in the standard assessment of hand function. These grips are assumed to be related to the skills required for touchscreen manipulation since they contribute to hand stability and fine motor coordination [22,23]. The grips assessed were: (1) hammer grip, defined as closing the hand with the thumb in opposition to all other fingers, (2) tip to tip pinch, defined as the index finger pressing against the thumb, and (3) three-point pinch (palmer pinch, three jaw chuck pinch), defined as pressing the thumb pulp against the index and middle fingers [22,23]. A calibrated JAMAR hand dynamometer (Sammons Preston Rolyan, Chicago, IL, USA) was used to measure hammer grip in accordance with the accepted seating and hand position, following the protocol adopted by the American Society of Hand Therapists [24]. JAMAR has excellent accuracy compared with known forces (*r* > 0.96) [25,26], good to excellent test-retest reliability (intraclass correlation coefficients [ICC] > 0.80) [27,28], and excellent result validity (ICC = 0.98–0.99) [29]. Tip to tip and three-point pinch were measured using the JAMAR hydraulic pinch gauge (Baseline, Fabrication Enterprises Inc., Irvingston, NY, USA). The system has excellent accuracy (±1%), excellent interrater reliability (ICC = 0.97) and excellent test-retest reliability (ICC > 0.8) [30]. Tests began with the dominant hand and proceeded in a fixed order. The measurements were repeated three times and means (in kg) were used for analysis. The literature contains several reports on the normative values for age, gender, and hand dominance [5,31,32,33].

#### 2.1.2. Manual Dexterity 

The FDT used to assess manual dexterity is a portable assessment tool, in which the individual is required to pick up 16 pegs in a predetermined order. After picking up each peg, the subject is asked to turn and replace it as quickly as possible in the same hole [12]. The tool was administered beginning with the dominant hand. The score (in seconds) was calculated, including time for completing the task and penalty seconds for mistakes. Higher scores indicated a lower manual dexterity [34]. Normative data for the FDT were reported in the literature based on examinee age, hand dominance, and occupation [12,34]. The FDT demonstrated excellent interrater reliability (ICC > 0.99) [12] and good construct validity (*r* value: 0.63–0.64) [12].

#### 2.1.3. Touchscreen Manipulation Ability 

The TATOO software application was used to determine touchscreen manipulation ability [20,35]. The touchscreen device was placed on a stable table in front of the seated subject. Beginning with the dominant hand, five tasks were employed: (1) touch all corners, (2) double tap, (3) drag in all directions, (4) drag along straight horizontal paths of varying lengths, and (5) pinch balloon (for further details see [35]). The tasks were performed in a fixed order, as determined by the software, and were repeated once following detailed verbal instructions and a demonstration. The duration of the entire test was approximately 15 min. Participants habitually using reading glasses were requested to use them while performing the tasks [35]. TATOO results provided information on the performance of different functional components required to use a touchscreen effectively. Performance of each task was summarized using numeric and graphic reports of the following parameters: (1) temporal, such as reaction time and duration time, and (2) accuracy, such as number of drag attempts successfully completed (for details see [21]).

### 2.2. Procedure

The enrolled participants were invited to a single 45 min session in which they were asked to perform, in a fixed order, a traditional hand function assessment, including hand grip strength, pinch strength, and the FDT, and the touchscreen manipulation ability test. 

### 2.3. Statistical Analyses

Descriptive statistics were used to present the characteristics of the study sample and the results of the hand function assessment. Correlation between the TATOO scores and the hand grip strength, pinch strength and the FDT was analyzed using Spearman’s correlation, as continuous parameters failed to meet the assumptions necessary for Pearson’s correlation. Strength of the relationship was determined using Cohen’s standard coefficient [36]. Correlation coefficients between 0.10 and 0.29 indicated a small effect, coefficients between 0.30 and 0.49 indicated a moderate effect, and coefficients ≥ 0.50 indicated a large effect. Accordingly, a coefficient threshold ≥ 0.3 was set as acceptable [36]. *p* values ≤ 0.05 were considered statistically significant. Statistical analyses were performed using SAS version 9.4 (SAS Institute, Cary, NC, USA).

## 3. Results

Fifty-four independent community-dwelling adults were enrolled to this study. 

Twenty individuals were excluded due to medical conditions that could affect hand function or cause severe pain or balance or gait impairments. Thirty-four with an average age of 79.4 ± 6.7 years (range, 65–95 years) participated in the study. All subjects reported being independent in basic and instrumental activities of daily living. Subject characteristics are presented in Table 1.

The results of the traditional hand function assessments are presented in Table 2.

Correlation results between TATOO parameters and traditional hand function outcome measures are presented in Table 3.

Of the 26 TATOO variables, only 4 were found to be significant: (1) the reaction time under the “Double Tap” was found to have moderate significant correlation with the tip to tip pinch (*r* value: 0.31, *p* value: 0.08); (2) the number of tips under the “Double Tap” was found to have moderate significant correlation with manual dexterity (*r* value: 0.32, *p* value: 0.07); (3) the number of drag attempts under the “Drag to different directions” task was found to have a moderate negative significant correlation with the power grip strength (*r* value: −0.39, *p* value:0.02), and (4) a moderate positive significant correlation with the FDT (*r* value: 0.36, *p* value: 0.04). The power of the correlation results was 66.84%.

## 4. Discussion

This study aimed to examine the correlation between the performance of elderly individuals while operating a tablet and the results of traditional hand assessment tools, such as grip and pinch strength and the FDT test. The results demonstrated no significant correlation between most of the items measuring touchscreen manipulation ability (22 out of 26) and traditional hand assessment tools. This suggests that the manual gestures necessary for the operation of a touchscreen entail unique and specific capabilities that are not captured by traditional tools of measuring hand function among the elderly. Therefore, touchscreen performance appears to be related to specific constructs of hand function, which need to be addressed.

However, four TATOO items demonstrated significant, yet moderate, correlations with the conventional assessments. The variable that tallied the number of drag attempts to different directions demonstrated a significant negative correlation with hand grip strength and a significant positive correlation with the FDT. This was the most complex TATOO task, as it required accurate application and maintaining pressure on clothing items while dragging them along varied paths to different locations on the screen. In contrast to other tasks, it required a single finger gesture, which involved dragging the item while simultaneously maintaining continuous pressure and releasing this pressure with precision at the end. Therefore, this task required finger dexterity, which may explain the significant negative correlation between the number of drag attempts and manual dexterity, where a higher number of attempts reflects lower dexterity. In addition, performing the gesture requires the ability to precisely apply pressure while maintaining hand stability, which may explain the correlation of this item with hand grip strength. Similarly, the correlation between the number of taps in “double taps” and manual dexterity, that was demonstrated through the analysis, may indicate that completing this task requires a degree of manual dexterity. The only correlation that we found difficult to explain is that between the reaction time of the “double tap” and the tip-to-tip pinch strength. This correlation should be further explored.

There are a number of reports indicting different normative values of hand grip strength among older adults [33,37]. These differences between reports likely reflect the differences between countries and cultures and factors such as nutrition, genetics, muscle mass, and the level of physical activity [33,38]. Hand grip strength in this study, which was conducted in Israel, was within the range of the reported data [33,37]. Hand grip strength is considered to be a strong indicator of low health-related outcomes [37]. Accordingly, The European Working Group on Sarcopenia in Older Persons (EWGSOP) determined cut off values for hand grip strength as 27 kg for men and 16 kg for women [38]. The results pertaining to hand grip strength in this study were higher than the recommended cut off values, indicating that the study sample included healthy elderly adults.

Different assessment tools are used to determine dexterity. Tests tend to focus on different components of dexterity, including hand steadiness, line tracking and tapping ability, and aiming accuracy [39]. A previous study [39] demonstrated that age and hand grip strength are predictors of hand dexterity among elderly adults with unequal impact on the various dexterity’s components. Steadiness and line tracking abilities were demonstrated to be moderated more by age than grip strength, while aiming and tapping seem to be correlated more with hand grip strength than age. Therefore, it was suggested that hand steadiness and line tracking ability depend to a great degree on muscle control and/or tremor rather them muscle strength [39]. The FDT, used in the present study, measures primarily steadiness and aiming abilities. Touchscreen manipulation may be similarly related to line tracking and tapping abilities, which may explain the lack of correlation with the FDT. Therefore, additional studies are required to evaluate the correlation between performance with the TATOO components of hand dexterity.

This study had some limitations. All participants were living independently within a community. Future studies should examine the correlation between TATOO performance and hand grip strength and manual dexterity in elderly populations with various impairments. This is supported by a previous study demonstrating correlations between tablet manipulation and measures of dexterity and cognitive level in patients following a stroke [40]. Furthermore, the present study performed assessments with the subjects in a sitting position and the tablet placed on the table at a comfortable distance from the subjects. Future studies should examine the ability to manipulate the tablet in more challenging positions, as common usage of tablets involves holding the device, which requires sufficient grip strength in addition to the screen manipulation [41].

## 5. Conclusions

The present study indicated that touchscreen manipulation ability among healthy elderly individuals 65 years of age or older was not correlated with grip and pinch strength and manual dexterity. Therefore, touchscreen performance appears to be related to specific constructs of hand function, which should be specifically addressed. The results have important clinical implications as they indicate that current traditional hand function assessments for the elderly do not address important constructs that are necessary for functioning in the modern technological world. Therefore, to evaluate the unique skills that are required to operate digital devices, new tools such as the TATOO must be included in the hand function assessment toolbox. However, the statistically moderate significant correlation between the number of drag attempts in the “Drag to different directions” task and hand grip strength and manual dexterity may indicate that more complex gestures require hand stability and accuracy, which are related to strength and dexterity. Further studies are required to explore the correlation between touchscreen manipulation ability, grip strength, and dexterity in different usage positions and with individuals with impairments.

## Figures and Tables

**Table 1:** Characteristics of the study sample (*n* = 34).

Variables	
Age in years, mean ± SD	79.4 ± 6.7
Gender, *n* (%) Male	11 (32.4)
Right Hand dominant, *n* (%)	34 (100)
Ownership of smartphone, *n* (%)	23 (67.6)

SD—standard deviation, *n*—number.

**Table 2:** Results of the traditional hand function assessment (Mean, SD, Median).

Variables	
Hand Grip Strength	
Dominant hand	23.3 ± 8.9, 20.8
Tip to tip Pinch (kg):	
Dominant hand	3.6 ± 2.4, 3.2
Three-point Pinch (kg):	
Dominant hand	4.8 ± 1.7, 4.7
Functional Dexterity Test (seconds):	
Dominant hand	45.6 ± 14, 44.7

**Table 3:** Correlation between the TATOO parameters and the traditional hand assessment of the right hand for older individuals (Spearman’s correlation coefficients, * *p*-value *≤* 0.05).

TATOO	Hand Grip Strength	Tip to Tip Pinch	Three-Point Pinch	Manual Dexterity
**Touch corners**				
Reaction time (s)	−0.3	−0.07	0.02	−0.05
Test duration (s)	0.14	0.09	0.16	−0.09
Flight time (s)	0.09	−0.12	0.006	−0.01
Touch time (s)	−0.08	0.01	0.0001	0.1
**Double tap**				
Reaction time (s)	−0.15	0.31 *	0.13	0.13
Test duration (s)	−0.06	0.18	0.04	0.19
No. of taps	0.006	0.07	−0.05	0.32 *
Correct Attempts (No)	0.22	0.04	0.04	0.08
Touches outside target (No)	0.1	0.12	0.008	0.31
Flight time (s)	−0.02	0.21	0.07	0.16
Touch time (s)	−0.18	−0.06	−0.1	0.23
**Drag to different directions**				
Reaction time (s)	−0.24	−0.1	−0.004	0.05
Test duration (s)	−0.24	−0.03	−0.08	0.13
Drag attempts (No)	−0.39 *	0.12	−0.16	0.36 *
Touches outside target (No)	−0.28	−0.01	−0.06	0.26
Flight time (s)	−0.26	−0.06	−0.03	0.2
Touch time (s)	−0.14	−0.004	−0.005	−0.06
**Drag along a horizontal path**				
Reaction time (s)	−0.18	−0.04	−0.13	0.08
Test duration (s)	−0.07	0.12	−0.04	0.29
Flight time (s)	−0.06	0.31	0.08	0.15
Touch time (s)	0.03	−0.02	−0.1	0.18
**Pinch ability**				
Reaction time (s)	0.02	0.006	−0.25	0.31
Test duration (s)	−0.19	−0.21	−0.25	0.12
Touches outside target (No)	−0.3	−0.25	−0.32	0.1
Flight time (s)	−0.19	−0.16	−0.31	0.29
Touch time (s)	−0.13	−0.11	−0.05	0.15

s—seconds; No—number.

## Data Availability

Data are available on request from the authors: michal.elboim@gmail.com and saadalexandra@gmail.com.
